# Beamforming Design for Full-Duplex SWIPT with Co-Channel Interference in Wireless Sensor Systems

**DOI:** 10.3390/s18103362

**Published:** 2018-10-08

**Authors:** Xiaoqing Liu, Yinglin Jia, Zhigang Wen, Junwei Zou, Shan Li

**Affiliations:** Beijing Key Laboratory of Work Safety Intelligent Monitoring, School of Electronic Engineering, Beijing University of Posts and Telecommunications, Beijing 100876, China; xq0723@bupt.edu.cn (X.L.); jiayl@bupt.edu.cn (Y.J.); buptzjw@bupt.edu.cn (J.Z.); lish01@ehualu.com (S.L.)

**Keywords:** amplify-and-forward relay system, co-channel interferers, full-duplex, imperfect channel state information, simultaneous wireless information and power transfer, successive convex approximation, wireless sensor networks

## Abstract

The simultaneous wireless information and power transfer (SWIPT) technique has been regarded as an appealing approach to prolong the lifetime of wireless sensor networks. However, co-channel interferences with SWIPT in wireless networks have not been investigated from a green communication perspective. In this paper, joint transmit and receive beamforming design for a full-duplex multiple-input multiple-output amplify-and-forward relay system with simultaneous wireless information and power transfer in WSNs is investigated. Multiple co-channel interferers are considered at the relay and destination sensor nodes. To minimize the mean-squared-error of the system, joint source and relay beamforming optimization is proposed while guaranteeing the transmit power constraints and destination’s energy harvesting constraint. An iterative algorithm based on alternating optimization with successive convex approximation which converges to a local optimum is proposed to solve the non-convex problem. Moreover, a low-complexity scheme is derived to reduce the computational complexity. Simulations for MSE versus iterations and MSE versus signal-to-noise ratio (SNR) demonstrate the convergence and good performance of the proposed schemes.

## 1. Introduction

A wireless sensor network (WSN), which consists of several low-cost sensor nodes performing sensing, computation and communication, is a paramount technique to gain and control information from surroundings such as building structures [1], the human body [2], surveillance [3], as well as anomaly detection [4,5]. However, WSNs are usually supplied by batteries with limited power [6], which will limit the amount of information for long distance transmission. For WSNs used for a special environment (for example, ecological monitoring), energy supply becomes one of the most important considerations compared with the traditional requirements on throughput, rates, delay and quality of service [7,8]. Moreover, massive MIMO is widely applied in WSNs to improve the energy efficiency [9,10,11].

Unlike the conventional energy supply techniques, which collect energy from natural sources such as wind, solar and thermal, simultaneous wireless information and power transfer (SWIPT) enables sensor receivers to decode information and scavenge energy from the same radio frequency signal, even in a hostile environment [12,13,14]. Thus, some researchers have paid attention to the SWIPT technologies and applications in WSNs considering the convenience and cost-effectiveness [15,16]. In [15], the secrecy performance of the two-user SWIPT sensor networks was studied, and a novel secure transmission scheme of cooperative zero-forcing (ZF) jamming was proposed. Joint resource optimization was investigated in an underlay cognitive sensor network (CSN) with an SWIPT-enabled amplify-and-forward (AF) relay node using the power splitting-based relaying (PSR) protocol in [16]. Two practical receiver architectures for multiple-input multiple-output (MIMO) broadcasting SWIPT, time switching (TS) and power splitting (PS), were discussed in [17], where a PS receiver structure splits the received signal into two different power streams, one for energy harvesting (EH) and the other for information decoding (ID).

Due to aggressive frequency reuse in wireless sensor networks, receivers suffer from co-channel interferers (CCIs). Systems with SWIPT operating in the presence of CCIs have been considered in [18,19,20,21,22]. The work in [18] considered a transmission scheme for a two-user MIMO interference channel (IFC) system with energy harvesting. The work in [19] extended the work in [18] to the *k*-user MIMO IFC case. In [20], energy harvesting (EH) in a decode-and-forward relaying system with CCIs was examined by analyzing ergodic capacity and transmission outage. In [21], an energy-harvesting amplify-and-forward (AF) relaying system corrupted by interference and Nakagami-*m* signal fading was investigated by deriving the outage probability and the throughput. The relaying SWIPT systems in [20,21] were extended in [22] by equipping each node with multiple antennas. Note that all work in [18,19,20,21,22] studied half-duplex (HD) mode.

Full-duplex (FD) mode has aroused an upsurge of interest since FD techniques achieve receiving and transmitting information simultaneously. The work in [23] considered an FD point-to-point (P2P) system with SWIPT, whose two nodes were equipped with two antennas, where transmit power and PS ratio were jointly optimized to maximize energy harvesting. A dual-hop FD relaying system with EH relay was investigated in [24], finding that FD relaying boosts the system throughput compared to conventional HD relaying. The work in [25] proposed an FD MIMO AF two-way relay system to maximize the achievable sum-rate under the assumption of perfect channel state information. The authors in [26] minimized the total mean-squared-error (MSE) of a FD MIMO AF relay system through beamforming design. Diversity and multiplexing gains are investigated and compared under half-duplex and full-duplex modes of cooperative systems in [27,28,29]. The transceiver design of full-duplex MIMO IoT devices with SWIPT was proposed in [30], and it showed that the EH technique can harvest enough energy to support power consumption-limited IoT devices by aiding in recharging their respective batteries. However, these existing works have not investigated CCIs with SWIPT in WSNs from a green communication perspective.

To overcome the above drawbacks, this paper focuses on a full-duplex MIMO AF relay SWIPT wireless sensor system, with the presence of CCIs on all receive antennas. Joint source-destination beamforming design and source-relay beamforming design are proposed to minimize the total MSE. The main contributions are described as below. First, compared with the systems in [20,21,22], whose relays operated in HD mode, and the systems in [23,24,25,26], where CCIs are ignored, our analysis is based on the FD mode considering multiple CCIs. Second, MSE criterion is used, minimized for the problem formulation, for its effectiveness in WSNs. Since the problem is non-convex and NP-hard, we propose an iterative algorithm based on alternating optimization (AO) with the successive convex approximation (SCA) method, which converges to a local optimum. Third, a closed-form suboptimal scheme is derived using the Lagrangian dual function and the Karush–Kuhn–Tucker (KKT) condition to reduce the computational complexity. Results for MSE versus iterations and MSE versus SNR demonstrate the convergence and the good performance of the proposed schemes.

The remainder of the paper is organized as follows. Section 2 characterizes the system including the deployment of sensor nodes and the optimization model. In Section 3, the iterative algorithms based on alternating optimization (AO) with SCA and Lagrangian methods are proposed. Numerical results and discussion are given in Section 4. Section 5 concludes the whole paper.

Notation: Throughout this paper, boldface uppercase letters are used to denote matrices, and lowercase letters denote vectors. ${\left(\cdot \right)}^{T}$ is the transpose operation, and ${\left(\cdot \right)}^{H}$ is the conjugate transpose operation. |·| is the bound norm of a matrix, and E[·] denotes the expectation operation.

## 2. System Model

This paper aims at jointly designing the transmitters of source and relay and the receivers of relay and destination in the FD relay wireless sensor system with SWIPT considering co-channel interference. In this system, we adopt multiple CCIs on all receive antennas and a PS receiver at the destination. Without loss of generality, we assume 100 percent energy transfer efficiency at the PS receiver and a fixed power splitting ratio for ID and EH. To clearly discuss such a problem, the node deployment and optimization model are described as follows for further analysis.

### 2.1. Node Deployment

**Figure 1:**
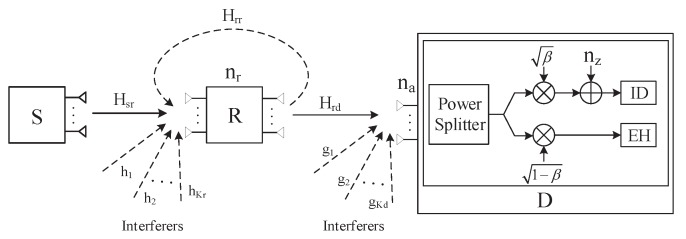
The simplified model of the FD MIMO SWIPT system with CCI.

The wireless FD MIMO AF relay SWIPT sensor system, consisting of a source node *S* with $M_{s}>1$ transmit antennas, a destination node *D* with $N_{d}>1$ receive antennas and an AF relay node *R* equipped with $M_{r}>1$ transmit antennas and $N_{r}>1$ receive antennas, is considered. *R* and *D* are subject to *M* CCIs and *N* CCIs, respectively, as depicted in Figure 1. *S* communicates with *D* via *R* where *D* receives information and harvests energy from *R* simultaneously. No direct link exists between *S* and *D*, and full channel state information (CSI) is available at each node.

To exploit spatial multiplexing for higher spectral efficiency, we assume $M_{s}$ data streams $s\left[m\right]\in C^{M_{s}\times 1}$ with normalized power are transmitted through the beamformer $F_{s}\in C^{M_{s}\times M_{s}}$ from *S* to *R* at time slot *m*. The signal $y_{r}\left[m\right]$ received by *R* is the combination of the desired signal from *S*, the CCIs’ signals and the loopback self-interference signal caused by using FD mode. Thus, $y_{r}\left[m\right]$ can be written as:

(1) $$\begin{array}{cc} y_{r}\left[m\right]= & H_{sr}F_{s}s\left[m\right]+\sum_{i=1}^{M}h_{i}x_{i}\left[m\right]+H_{rr}x_{r}\left[m\right]+n_{r}\left[m\right] \end{array}$$

where $H_{sr}\in C^{N_{r}\times M_{s}}$ and $H_{rr}\in C^{M_{r}\times N_{r}}$ denote the channel gains from *S* to *R* and the self-interference channel at *R*, respectively, $h_{i}\in C^{N_{r}\times 1}$ is the *i*-th interference channel gain, $x_{i}$ is the *i*-th interferer signal at *R*, $x_{r}$ is the signal transmitted by *R*, which is regarded as self-interference by receivers at *R*, and $n_{r}∼\left(0,\sigma_{r}^{2}I_{M_{r}}\right)$ represents additive white Gaussian noise (AWGN) at *R*. All channels are assumed to be mutually independent and frequency flat. Let $H_{I}=\left[h_{1},h_{2},\ldots ,h_{M}\right]\in C^{N_{r}\times M}$ be the CCIs’ gain matrix at the relay, and let $x_{I}={\left[x_{1},x_{2},\ldots ,x_{M}\right]}^{T}$ be the interferer signal vector with the total interference power constraint ${∥x_{I}∥}^{2}=p_{1}$. Replace term $\sum_{i=1}^{M}h_{i}x_{i}\left[m\right]$ in Equation (1) by $H_{I}x_{I}\left[m\right]$.

In practice, the signal transfer at the relay will cause a τ(≥1)-symbol processing delay. Since the receiver of *R* has knowledge of the signal transmitted by the transmitter of *R*, the self-interference is assumed to be canceled perfectly or almost perfectly [22]. The near-negligible residual self-interference can be regarded as an additional relay input noise [23], whereupon the signal $x_{r}\left[m\right]$ can be expressed as:

(2) $$\begin{array}{cc} x_{r}\left[m\right] & =F_{r}\left(y_{r}\left[m-\tau \right]-H_{rr}x_{r}\left[m-\tau \right]\right)\\ & =F_{r}\left(H_{sr}F_{s}s\left[m-\tau \right]+H_{I}x_{I}\left[m-\tau \right]+n_{r}\left[m-\tau \right]\right) \end{array}$$

where $F_{r}\in C^{N_{r}\times M_{r}}$ denotes the transmit beamforming matrix of *R*. The signal received at *D* becomes:

(3) $$y_{d}\left[m\right]=H_{rd}x_{r}\left[m\right]+\sum_{i=1}^{N}g_{i}y_{i}\left[m\right]+n_{a}\left[m\right]$$

where $H_{rd}\in C^{N_{s}\times M_{r}}$ and $g_{i}$ denote the channel gains from *R* to *D* and the *i*-th interference channel at the destination and $y_{i}$ is the *i*-th interferer signal at *D*. Let $G_{I}=\left[g_{1},g_{2},\ldots ,g_{N}\right]\in C^{N_{d}\times N}$ be the CCIs’ gain matrix at the destination, and let $y_{I}={\left[y_{1},y_{2},\ldots ,y_{N}\right]}^{T}$ be the interferer signal vector with total interference power constraint ${∥y_{I}∥}^{2}=p_{2}$, then Equation (3) can be rewritten as:

(4) $$y_{d}\left[m\right]=H_{rd}x_{r}\left[m\right]+G_{I}y_{I}\left[m\right]+n_{a}\left[m\right].$$

Define the equivalent noise vector:

(5) $$n_{d}\left[m\right]=\sqrt{\beta }n_{a}\left[m\right]+n_{z}\left[m\right]$$

where $n_{z}\left[m\right]$ is the AWGN originating from the power splitter and $n_{d}\left[m\right]$ represents the overall noise at *D* with zero mean and covariance matrix $\sigma_{d}^{2}I_{N_{d}}$. The received signal at *D* is split into two parts under the PS protocol, where a portion, $\beta \in \left(0,1\right)$, of power is allocated for information decoding (ID). Thus, the signal for ID is:

(6) $$y_{id}\left[m\right]=\sqrt{\beta }\left(H_{rd}x_{r}\left[m\right]+G_{I}y_{I}\left[m\right]\right)+n_{d}\left[m\right].$$

Since memoryless channels are assumed in this system, we omit the time index to have $s≜s\left[m-\tau \right]$, $x_{r}=x_{r}\left[m\right]$, $x_{I}≜x_{I}\left[m-\tau \right]$, $n_{r}≜n_{r}\left[m-\tau \right]$, $y_{I}≜y_{I}\left[m\right]$ and $n_{r}≜n_{r}\left[m\right]$. Then, Equation (6) can be simplified as:

(7) $$y_{id}=\sqrt{\beta }\left(H_{rd}x_{r}+G_{I}y_{I}\right)+n_{d}.$$

### 2.2. Optimization Model

Considering both transmit power and receive power constraints, this section presents an optimization model to obtain the optimal beamforming scheme minimizing the MSE of the whole system. The beamforming scheme includes the transmit beamformer $F_{s}$ at *S*, $F_{r}$ at *R* and the linear receiver at *D*. Both the objective and constraints are discussed as follows.

The MSE of the signal at *D* can be calculated as:

(8) $$\begin{array}{cc} \mathrm{MS} & E=E\left[{∥W_{d}y_{id}-s∥}^{2}\right]\\ = & \mathrm{Tr}\{\beta W_{d}H_{rd}F_{r}RF_{r}^{H}H_{rd}^{H}W_{d}^{H}\\ - & \sqrt{\beta }F_{s}^{H}H_{sr}^{H}F_{r}^{H}H_{rd}^{H}W_{d}^{H}-\sqrt{\beta }W_{d}H_{rd}F_{r}^{H}H_{sr}F_{s}\\ + & p_{2}\beta W_{d}G_{I}G_{I}^{H}W_{d}^{H}+\sigma_{d}^{2}W_{d}W_{d}^{H}+I_{M_{s}}\} \end{array}$$

where $R=H_{sr}F_{s}F_{s}^{H}H_{sr}^{H}+p_{1}H_{I}H_{I}^{H}+\sigma_{r}^{2}I_{N_{r}}$ and $W_{d}$ is the linear receiver at *D*. Define $p_{s}$ and $p_{r}$ as the peak power thresholds of *S* and *R*. The transmit power at *S* and transmit power at *R* should satisfy the following constraints:

(9) $$\mathrm{Tr}\left(F_{s}F_{s}^{H}\right)\leq p_{s}$$

(10) $$\mathrm{Tr}\left(F_{r}RF_{r}^{H}\right)\leq p_{r}.$$

A (1−β) portion of the signal received at *D* is used for EH, and the power harvested at *D* should surpass the minimum power threshold:

(11) $$\left(1-\beta \right)\mathrm{Tr}\left(H_{rd}F_{r}RF_{r}^{H}H_{rd}^{H}+p_{2}G_{I}G_{I}^{H}\right)\geq e.$$

Under the MSE criterion, the optimal joint source and relay beamforming problem with power constraints can be formulated as:

(12) $$\min_{W_{d},\;F_{r},\;F_{s}}\;\mathrm{MSE}\;\;\;\;\;$$

(13) $$s.t.\;\mathrm{Tr}\left(F_{s}F_{s}^{H}\right)\leq p_{s}\;\;\;\;$$

(14) $$\mathrm{Tr}\left(F_{r}RF_{r}^{H}\right)\leq p_{r}\;$$

(15) $$\left(1-\beta \right)\mathrm{Tr}\left(H_{rd}F_{r}RF_{r}^{H}H_{rd}^{H}+p_{2}G_{I}G_{I}^{H}\right)\geq e.$$

## 3. Scheme Design

Since the problem Equation (12) is multi-variate and non-convex, an iterative algorithm based on alternating optimization (AO) is employed where in each iteration, each variable is optimized alternatively with the others fixed. Then, the original optimization problem is divided into three subproblems.

First, with $F_{r}$ and $F_{s}$ fixed, the linear receiver $W_{d}$ is optimized. Since there is no $W_{d}$ involved in any constraint of problem Equation (12), the optimal $W_{d}^{opt}$ can be derived using the Wiener filtering principle $\partial \mathrm{MSE}/\partial W_{d}^{*}=0$, which yields:

(16) $$\begin{array}{c} W_{d}^{opt}=\sqrt{\beta }F_{s}^{H}H_{sr}^{H}F_{r}^{H}H_{rd}^{H}{\left(\beta H_{rd}F_{r}RF_{r}^{H}H_{rd}^{H}+p_{2}\beta G_{I}G_{I}^{H}+\sigma_{d}^{2}I_{M_{s}}\right)}^{-1}. \end{array}$$

In the following subsection, the optimization of $F_{r}$ and $F_{s}$ is discussed.

### 3.1. Optimization of $F_{r}$

With $F_{s}$ and $W_{d}$ fixed, $F_{r}$ is first optimized. According to [11], p. 77:

(17) $$\mathrm{Tr}\left(\mathrm{ABCD}\right)={\left(vec(D^{T})\right)}^{T}\left(C^{T}⊗A\right)vec\left(B\right)$$

where A, B, C and D are arbitrary matrices with compatible dimensions, vec(·) denotes the matrix vectorization operator and the symbol ⊗ signifies the Kronecker product. By applying the result from Equation (17) to problem Equation (12), it can be equivalently transformed into:

(18) $$\min_{f_{r}}\;f_{r}^{H}Q_{r}f_{r}-f_{r}^{H}q_{r}-q_{r}^{H}f_{r}+C_{r}\;$$

(19) $$s.t.\;\;f_{r}^{H}Q_{r1}f_{r}\leq p_{r}\;\;\;\;\;\;\;$$

(20) $$f_{r}^{H}Q_{r2}f_{r}\leq \frac{e}{1-\beta }+p_{2}\mathrm{Tr}\left(G_{I}G_{I}^{H}\right)$$

where $f_{r}=vec\left(F_{r}\right)$, $Q_{r}=R^{T}⊗\left(\beta H_{rd}^{H}W_{d}^{H}W_{d}H_{rd}\right)$, $C_{r}=\mathrm{Tr}\left(p_{2}\beta W_{d}G_{I}G_{I}^{H}W_{d}^{H}+\sigma_{d}^{2}W_{d}W_{d}^{H}+I_{M_{r}}\right)$, $q_{r}=vec\left(\sqrt{\beta }H_{rd}^{H}W_{d}^{H}F_{s}^{H}H_{sr}^{H}\right)$, $Q_{r1}=R^{T}⊗I_{M_{r}}$, and $Q_{r2}=-R^{T}⊗\left(H_{rd}^{H}H_{rd}\right)$. Note that matrix $Q_{r2}$ is negative semidefinite, and thus, constraint Equation (20) is non-convex. To tackle the non-convexity of this subproblem, the SCA method is proposed. Defining $g\left(f_{r}\right)≜f_{r}^{H}Q_{r2}f_{r}$ and using a center point $z_{r}=C^{N\times 1}$, where $N=N_{r}\times M_{r}$, $\hat{g}$ is defined as:

(21) g^(fr)≜2RezrHQr2fr−zrHQr2zr.

By exploring the properties of g and $\hat{g}$, we can obtain the following Property 1.

**Property** **1.** *$\hat{g}$ has the following properties:*

 *(i)* *$\hat{g}\left(z_{r}\right)=g\left(z_{r}\right)$;*
 *(ii)* *$\hat{g}\left(f_{r}\right)\geq g\left(f_{r}\right)$;*
 *(iii)* *$\partial \hat{g}/\partial f_{r}{{|}_{f_{r}=z_{r}}=\partial g/\partial f_{r}|}_{f_{r}=z_{r}}$.*

**Proof.**  Please refer to Appendix A. ☐

Replacing g with $\hat{g}$, subproblem Equation (18) can be approximated as:

(22) $$\min_{f_{r}}\;f_{r}^{H}Q_{r}f_{r}-f_{r}^{H}q_{r}-q_{r}^{H}f_{r}+C_{r}$$

(23) $$s.t.\;\;\;f_{r}^{H}Q_{r1}f_{r}\leq p_{r}\;\;\;\;$$

(24) 2RezrHQr2fr≤zrHQr2zr+ρr

where $\rho_{r}=\frac{e}{1-\beta }+p_{2}\mathrm{Tr}\left(G_{I}G_{I}^{H}\right)$. Problem Equation (22) is convex and can be easily formulated as a second-order cone programming (SOCP) [31], which can be efficiently solved by SeDuMi. Denote the solution of Equation (22) as $f_{r}^{⋆}$. Then, update the new center point $z_{r}=f_{r}^{⋆}$, and repeat the process for the next iteration. Such an SCA-based iterative procedure is convergent since SCA converges to a KKT point [32].

### 3.2. Optimization of $F_{s}$

By fixing $F_{r}$ and $W_{d}$, the optimization of $F_{s}$ can be transformed into:

(25) $$\min_{f_{s}}\;f_{s}^{H}Q_{s}f_{s}-f_{s}^{H}q_{s}-q_{s}^{H}f_{s}+C_{s}$$

(26) $$s.t.\;\;\;\;f_{s}^{H}f_{s}\leq p_{s}\;\;\;\;$$

(27) $$f_{s}^{H}Q_{s1}f_{s}\leq \rho_{s1}$$

(28) $$f_{s}^{H}Q_{s2}f_{s}\leq \rho_{s2}$$

where $f_{s}=vec\left(F_{s}\right)$, $\Theta =H_{rd}F_{r}$, $\psi =F_{r}H_{sr}$, $\Gamma =p_{1}H_{I}H_{I}^{H}+\sigma_{r}^{2}I_{M_{r}}$, $q_{s}=vec\left(\sqrt{\beta }H_{sr}^{H}\Theta^{H}W_{d}^{H}\right)$, $Q_{s}=I_{M_{s}}^{T}⊗\left(\beta \psi H_{rd}^{H}W_{d}^{H}W_{d}H_{rd}\psi \right)$, $Q_{s1}=I_{M_{s}}⊗\left(\psi^{H}\psi \right)$, $Q_{s2}=-I_{M_{s}}⊗\left(\psi^{H}H_{rd}^{H}H_{rd}\psi \right)$, $\rho_{s1}=p_{r}-\mathrm{Tr}\left(F_{r}\Gamma F_{r}^{H}\right)$, $C_{s}=\mathrm{Tr}\left(\beta W_{d}\Theta \Gamma \Theta^{H}W_{d}^{H}+p_{2}\beta W_{d}G_{I}G_{I}^{H}W_{d}^{H}+\sigma_{d}^{2}W_{d}W_{d}^{H}+I_{M_{s}}\right)$ and $\rho_{s2}=\frac{e}{\beta -1}+\mathrm{Tr}\left(\Theta \Gamma \Theta^{H}+p_{2}G_{I}G_{I}^{H}\right)$. Since $Q_{s2}$ is negative, Problem (25) is non-convex. It can be solved using analysis similar to that used to solve for $F_{r}$, and the details are omitted here.

### 3.3. Alternating Optimization Algorithm

An iterative algorithm based on the procedure solving $W_{d}$, $F_{r}$ and $F_{s}$ alternatively is summarized as Algorithm 1 below.

**Algorithm 1** Alternating optimization algorithm based on SCA.**1. Initialize** Set β=0.6 and the initial beamforming matrix $F_{s}=\sqrt{\frac{p_{s}}{M_{s}}}I_{M_{s}}$, $F_{r}=\sqrt{\frac{p_{r}}{\mathrm{Tr}(R)}}I_{M_{r}}$.
**2. Repeat**
(1)Update $F_{r}$, w.r.t. $F_{s}$, $W_{d}$.
Set $k_{r}=0$ and the initial center point $z_{r}^{0}=vec\left(F_{r}\right)$.Solve problem Equation (22) at the $k_{r}$-th iteration to obtain $F_{r}^{k_{r}}$ when $k_{r}\geq 0$.Then, set $z_{r}^{k_{r}+1}=vec\left(F_{r}^{k_{r}}\right)$ and $k_{r}=k_{r}+1$.Until ${∥z_{r}^{k_{r}+1}-z_{r}^{k_{r}}∥}^{2}\leq 10^{-4}$, and let $F_{r}=mat\left(z_{r}^{k_{r}+1}\right)$.(2)Update $F_{s}$ with $k_{s}$ iterations, w.r.t. $F_{r}$, $W_{d}$, following similar steps to those in (1).(3)Update $W_{d}$, w.r.t. $F_{r}$, $F_{s}$.

**3. Until ▵MSE≤δ or $iter\geq iter_{max}$**


Notice that Algorithm 1 is convergent according to the following Property 2.

**Property** **2.** *The proposed alternating optimization algorithm based on SCA is convergent.*

**Proof.**  Please refer to Appendix B. ☐

Since Equation (16) is in closed-form, the total complexity of the proposed SCA-based Algorithm 1 is $O[k_{AO}\left(k_{r}M_{r}^{3.5}N_{r}^{3.5}+k_{s}M_{s}^{7}\right)]$, where $k_{AO}$ denotes the number of iterations needed for the alternating optimization to converge. Therefore, Algorithm 1 is of high complexity, and we therefore propose a low-complexity design for $F_{r}$ in the following subsection.

### 3.4. Low-Complexity Design for $F_{r}$

Since there are two constraints in problem Equation (18), we replace the inequality constraint Equation (19) by an equality constraint, thus tightening the feasible region. Then, introduce $\xi =\frac{\varepsilon }{p_{r}}$, where $\varepsilon =\frac{e}{1-\beta }+p_{2}\mathrm{Tr}\left(G_{I}G_{I}^{H}\right)$, and a matrix U composed of the negative eigenvalues of the matrix $Q_{r2}-\xi Q_{r1}$, and let $f_{r}=Ux$. Clearly, $U^{H}\left(Q_{r2}-\xi Q_{r1}\right)U$ is negative semidefinite, and the constraint $x^{H}U^{H}\left(Q_{r2}-\xi Q_{r1}\right)Ux\leq 0$ is satisfied. Thus, Equation (18) can be converted into:

(29) $$\Gamma =x^{H}U^{H}Q_{r}Ux-x^{H}U^{H}q_{r}-q_{r}^{H}Ux+C_{r}.$$

The optimization problem can be reduced to:

(30) $$\min_{x}\;\;\;\;\;\;\Gamma \;\;\;$$

(31) $$s.t.\;x^{H}U^{H}Q_{r1}Ux=p_{r}.$$

The Lagrangian function is formulated as:

(32) $$\begin{array}{cc} L=x^{H}U^{H}Q_{r}Ux & -x^{H}U^{H}q_{r}-q_{r}^{H}Ux+C_{r}\\ & +\mu (x^{H}U^{H}Q_{r1}Ux-p_{r}) \end{array}$$

where μ is the Lagrange multiplier and x satisfies the KKT condition. Solve the equation $\partial L/\partial x^{*}=0$, and then, the optimal $f_{r}$ can be obtained by the optimal $x^{opt}$ given by:

(33) $$x^{opt}={\left(U^{H}Q_{r}U+\mu U^{H}Q_{r1}U\right)}^{-1}U^{H}q_{r}$$

where $\mu \in (0,\sqrt{\frac{q_{r}^{H}{\left(Q_{r1}^{H}\right)}^{-1}q_{r}}{p_{r}}})$. μ can be obtained using the bisection search method. The computational complexity of the whole algorithm can be calculated as $O(k_{AO}\left(k_{r}M_{r}^{3.5}N_{r}^{3.5}+log\left(N_{r}\cdot M_{r}\right)\right)$, which is much lower than that of Algorithm 1.

## 4. Numerical Results and Discussion

In this section, we use Monte Carlo (MC) simulations to evaluate the performances of the proposed algorithms using QPSK modulation in [33]. Independent random Rayleigh fading channels ∼CN(0,1) with 100 slots were generated as operated in [34]. Set β=0.5, and the variances of the AWGNs were set as $\sigma_{n1}^{2}=\sigma_{n2}^{2}=\sigma_{n3}^{2}=\sigma^{2}$, $\sigma_{r}^{2}=\sigma_{d}^{2}=\sigma^{2}$. Then, SNR = $10\;log_{10}\left(E_{s}/\sigma^{2}\right)$. Let $E_{s}$ denote unit power, then $p_{1}=10E_{s}$, $p_{2}=E_{s}$, $p_{s}=10E_{s}$, $p_{r}=4E_{s}$, $p_{I1}=p_{I2}={p^{*}}_{I1}={p^{*}}_{I2}=0.7E_{s}$, $\sigma_{errH_{1}}^{2}=\sigma_{errH_{2}}^{2}=\sigma_{errH_{j1}}^{2}=\sigma_{errH_{j2}}^{2}=\sigma_{errG_{1}}^{2}=\sigma_{errG_{2}}^{2}=\sigma_{err}^{2}$. Three schemes are proposed for comparison here. Scheme 1 is the unaided scheme with initialized values and hence has no iterations. Scheme 2 is the SCA-based scheme, and Scheme 3 is the low-complexity suboptimal scheme.

**Figure 2:**
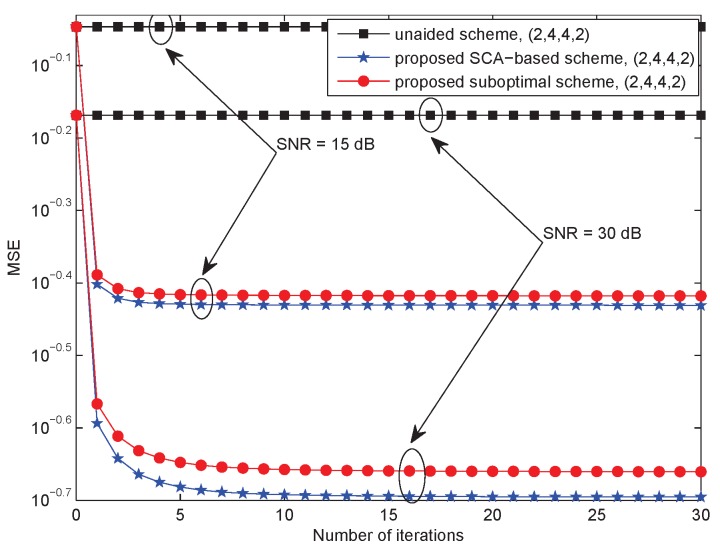
AF relay system: The MSE as a function of the number of iterations for {2,4,4,2}.

In Figure 2, let $\left\{M_{s},N_{r},M_{r},N_{d}\right\}\in \left\{2,4,4,2\right\}$, $K_{r}=K_{d}=2$ and SNR ∈{15,30} dB. Curves of MSE versus the number of iterations indicate that the algorithms were all convergent. We found that convergence to the MSE floor was rapid. When SNR = 15 dB, the proposed SCA-based scheme and suboptimal scheme were both convergent after six iterations. However, when SNR = 30 dB, the proposed SCA-based scheme did not achieve convergence until 20 iterations, while the suboptimal scheme became convergent after 10 iteration. Therefore, the conclusion that the low-complexity design proposed in Section 3.4 decreases the computational complexity of the SCA-based algorithm proposed in Section 3.3 was verified.

**Figure 3:**
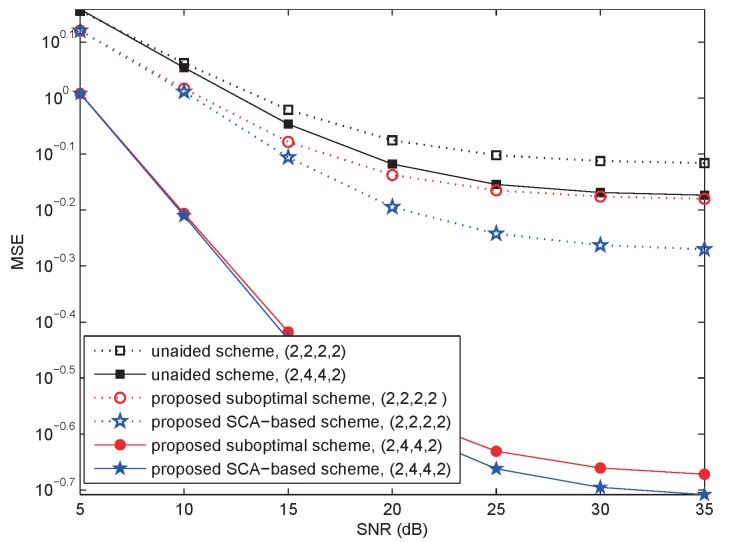
AF relay system: The MSE as a function of SNR for 30 iterations.

In Figure 3, we analyze the performance of the MSE versus SNR when $\left\{M_{s},N_{r},M_{r},N_{d}\right\}\in \left\{2,4,4,2\right\}$ and {2,2,2,2} separately. Set $K_{r}=K_{d}=2$. As expected, both when the antenna number of relay equaled two and four, the MSE of the proposed SCA-based scheme was the lowest, which indicates that it outperformed other schemes for MSE, and the unaided scheme was the worst. The second best was the proposed suboptimal scheme.

However, the performance difference was substantially reduced when diversity was used at the relay, so that the reduced complexity of the suboptimal scheme could make it the preferred choice. For example, when there was a four-fold transmitter and receiver diversity at the relay, the performances of the SCA-based and suboptimal schemes were virtually the same, as seen in the solid lines of Figure 3. Meanwhile, when the relay diversity order was small, the SCA-based scheme showed larger performance gain over the suboptimal scheme, as seen in the dotted lines of Figure 3. For example, when the relay diversity order was two, the MSE floor was reduced from 0.66 to 0.54 by using the SCA-based scheme. The performance gain achieved by using the SCA-based scheme over the suboptimal scheme can be evaluated for different combinations of transmitter and receiver diversity using the theory and results of this paper.

**Figure 4:**
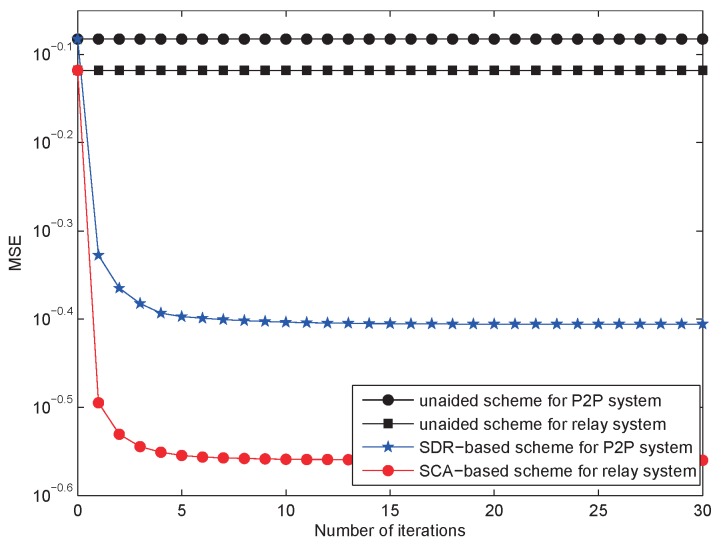
The comparison between the AF relay system and the P2P system for SNR = 20 dB.

Figure 4 is the comparison between the AF relay system and FD point-to-point system with co-interference on both nodes, as well as the proposed SCA-based scheme and the existing SDR-based scheme proposed in [35]. Comparing the unaided schemes, we can find that the MSE of the system with relay was lower than that of point-to-point system, which implies the advantage of relays. When the number of iteration was 30, the MSE of the unaided scheme for the relay system was $10^{-0.118}$, 7.84% lower than that of the point-to-point system. Meanwhile, the MSE of the proposed SCA-based scheme was $10^{-0.5599}$, 29.92% lower than that of the SDR-based scheme. It can be noticed that the gap between the SCA-based scheme and the SDR-based scheme was much larger than that of the two unaided schemes. Thus, we can make the conclusion that our proposed SCA-based scheme performed better than the existing SDR-based scheme.

## 5. Conclusions

An FD MIMO AF relay SWIPT wireless sensor network corrupted by co-channel interference was analyzed and optimized to minimize the total MSE. We derived an SCA-based scheme and a proposed low-complexity scheme that jointly optimized the source and relay beamforming matrices and linear receiver. While the SCA-based scheme exhibits superior performance, the low-complexity scheme offers virtually the same MSE performance with reduced complexity when four-fold diversity is employed at the relay. All of the findings are of great importance in guiding the beamforming design of practical wireless sensor networks with SWIPT. In future work, we plan to investigate the systems with multiple uses, to improve our algorithm by combining with other methods and to extend our approach to time-varying channels.

## Abbreviations

The following abbreviations are used in this manuscript:

WSN	Wireless sensor network
SWIPT	Simultaneous wireless information and power transfer
ZF	Zero-forcing
CSN	Cognitive sensor network
AF	Amplify-and-forward
PSR	Power splitting-based relaying
MIMO	Multiple-input multiple-output
TS	Time switching
PS	Power splitting
EH	Energy harvesting
ID	Information decoding
CCIs	Co-channel interferers
IFC	Interference channel
HD	Half-duplex
FD	Full-duplex
P2P	Point-to-point
MSE	Mean-squared-error
SCA	Successive convex approximation
AO	Alternating optimization
KKT	Karush–Kuhn–Tucker
CSI	Channel state information
AWGN	Additive white Gaussian noise
SOCP	Second-order cone programming

## Appendix A

Substituting $z_{r}$ into the definition of $\hat{g}$ and g Equation (21), Part (i) can be proven. Since $Q_{r2}$ is negative semidefinite, the inequality:

(A1) $${\left(f_{r}-z_{r}\right)}^{H}Q_{r2}\left(f_{r}-z_{r}\right)\leq 0$$

always holds, which implies:

(A2) frHQr2fr≤2RezrHQr2fr−zrHQr2zr.

Part (ii) is thus verified. As for Part (iii), by calculating the derivatives of $\hat{g}$ and g with respect to $f_{r}$, we have:

(A3) $$\partial \hat{g}/\partial f_{r}={\left(z_{r}^{H}Q_{r2}\right)}^{T},\;\partial g/\partial f_{r}={\left(f_{r}^{H}Q_{r2}\right)}^{T}.$$

Then, putting $z_{r}$ into Equation (A3), Part (iii) is proven.

## Appendix B

In the *k*-th iteration of the AO algorithm, we first calculate $F_{r}^{\left[k\right]}$ with the fixed $F_{s}^{\left[k-1\right]}$ and $W_{d}^{\left[k-1\right]}$. Since the optimal solution $f_{r}^{\left[k\right]}$ can be achievable with CVX, we can obtain that the objective value corresponding to $F_{r}^{\left[k\right]}$, $F_{s}^{\left[k-1\right]}$ and $W_{d}^{\left[k-1\right]}$ is no larger than that to $F_{r}^{\left[k-1\right]}$, $F_{s}^{\left[k-1\right]}$ and $W_{d}^{\left[k-1\right]}$. Similarly, the $F_{s}^{\left[k\right]}$ is no larger than that to $F_{s}^{\left[k-1\right]}$; $W_{d}^{\left[k\right]}$ is optimally solved, and the objective value is descendent. Consequently, the objective value of the original problem monotonically decreases and is lower bounded by zero, which testifies to the convergence of our proposed algorithm.
